# Negotiating familial mental illness stigma: The role of family members of persons living with mental illnesses

**DOI:** 10.1371/journal.pone.0311170

**Published:** 2024-09-30

**Authors:** Joseph Adu, Abram Oudshoorn, Kelly Anderson, Carrie Anne Marshall, Heather Stuart

**Affiliations:** 1 Department of Health and Rehabilitation Sciences, Elborn College, Western University, London, Ontario, Canada; 2 Arthur Labatt Family School of Nursing, Western University, London, Ontario, Canada; 3 Department of Epidemiology and Biostatistics, Schulich School of Medicine and Dentistry, Western University, London, Ontario, Canada; 4 School of Occupational Therapy, Faculty of Health Sciences, Western University, London, ON, Canada; 5 Department of Public Health Sciences, Department of Psychiatry and the School of Rehabilitation Therapy, Queens University, Kingston, Ontario, Canada; University of Sharjah College of Health Sciences, UNITED ARAB EMIRATES

## Abstract

**Background:**

This study explores how family members of individuals with mental illnesses address potential familial mental illness stigma. Previous studies have concentrated on self, social, and associative stigma and its impacts on families and persons with mental illnesses. Far less work has considered family members as perpetrators of mental illness stigma towards their loved ones with mental illnesses.

**Methodology/Principal findings:**

We conducted this study with 15 participants who were family members of persons with mental illnesses using semi-structured qualitative interviews. The in-depth interviews were followed by inductive analysis using Braun and Clarke’s technique for thematic analysis. Participants’ views on familial mental illness stigma and ways to reduce this were reported in five key themes. The themes included: (1) layered perspectives of social and family stigma; (2) family-related stigma; (3) complex interplay of family relationships and mental illness; (4) confronting stigma personally; and (5) envisioning a better future. The uncertainties connected with mental illnesses and the increased social stigma were conceptualized as contributors to familial mental illness stigma as ways to prevent potential associative stigma.

**Conclusion/Significance:**

Participants suggested the need for more social contact-based education and positive media reporting to correct the ongoing fallacies around mental illnesses. This study highlights how higher-order reforms to social systems and services would support both families and those living with mental illnesses to have more positive experiences.

## Introduction and purpose

Untreated mental illness leads to significant morbidity and mortality outcomes [1, 2]. In Canada, mental illnesses have been found to contribute significantly to disability, affecting 1 in every 5 people [3, 4]. Globally, over 70% of persons with mental illnesses do not have access to treatment, and much of this is attributed to stigma [5–7]. There is high social vulnerability and marginalization linked to mental illnesses [8, 9]. Such social vulnerabilities can make life complex for individuals with mental illnesses, diagnosed or otherwise, as well as their immediate families, especially in situations where lack of access to mental healthcare services makes families struggle to meet basic health needs while searching for treatment for their loved ones [10, 11]. In this study persons with mental illnesses and individuals with mental illnesses will be used interchangeably.

Individuals with mental illnesses often encounter multifaceted problems due to the ongoing stigma that is associated with their illnesses [12–14]. Goffman was one of the first to theoretically unpack stigma, describing stigma as a social construct that transmits stereotypes, which are deeply rooted in society. According to Goffman, such negative stereotypes lead to dishonor and avoidance of the person’s (race, ethnicity, and religion), physical deformities (e.g., deafness and blindness), and undesirable attributes (e.g., addiction and mental health problems) [15]. However, it is noted that the terminology used by Goffman represents discriminations of that time, such as labelling certain things as undesirable attributes. The social devaluation that the stigmatized person experiences reduces their full ability to participate in social life in their community [15]. As much as mental illnesses are regarded as a personal problem Malla and colleagues [16], concluded that mental health problems are a familial experience as well, given that most regular support for individuals with mental illnesses is provided by immediate family members or partners [17, 18]. Global research identifies mental illness stigma as a key contributor to the vulnerability and social exclusion of persons with mental illnesses within their neighborhood or immediate environment [14, 18–20].

The stigma associated with mental illness has been widely studied [10, 21–23]. Nevertheless, many of these studies explored stigma from the public or mental health professionals [24–27]. Other studies have also focused on stigma by association, (i.e., stigma experienced by health professionals or the ill person’s close family members) [28–34]. What is very limited in the literature is consideration of the stigma perpetrated by the individual’s family members (familial mental illness stigma) towards their relative who has been diagnosed with a mental illness. The paucity of literature on familial mental illness stigma may limit tools in place to address this stigma within the family system.

Research has acknowledged that some individuals are stigmatized by family members, which results in emotional distress and a lack of practical support [35–37]. Experiences of discrimination and humiliation within the family system may compel affected individuals to conceal their mental health problems from close relatives, thereby hampering ongoing treatment and social support for these individuals [26, 35, 36, 38]. A thorough understanding of familial mental illness stigma is crucial due to the potential existence of exclusionary behaviors towards persons with mental illnesses by their own families [23, 39].

The complex role of family members in relation to both social support and perpetration of stigma has been explored among relatives of persons diagnosed with schizophrenia, including the lived experience of parents involved in caring for young people experiencing a first episode of psychosis [40–43]. Though some of these studies focused on family experiences of mental illness-related stigma, there is still a lack of focus on the stigma that originates from within the family towards close relatives with mental illness. The role of the family in supporting relatives with mental illnesses in relation to social and emotional assistance is evident in the extant literature [23, 44–47]. This notwithstanding, it is prudent for researchers to ponder on stigma enacted by some family members towards their close relatives with mental illnesses due to the multi-faceted nature of mental illness stigma.

Familial mental illness stigma occurs when family members or close relatives enact stigma through labelling, stereotyping, prejudice, and discrimination against a family member with mental illnesses. In Canada, about 11 million (38%) Canadians aged 15 and above have at least one immediate or extended family member who lives with a mental health concern, diagnosed or otherwise [49, 50]. Of Canadian families with a loved one living with a mental health concern, nearly 71% of them felt that their own livelihoods were impeded by their relatives’ illness [48–50]. Just like the other forms of stigma, familial mental illness stigma could also be multi-faceted, warranting special attention in global research for scholars and practitioners to understand the unique experiences of both patients and family members. In this way, we could develop interventions to support families in addressing the stigma they knowingly or unknowingly may perpetrate against their relatives. This current study, therefore, explores how family members of persons living with mental illnesses address potential familial mental illness stigma in Ontario, Canada.

The objective of the study is to help fill some notable gaps about familial mental illness stigma, contribute to the anti-stigma literature, and promote evidence-based practices and policies towards stigma reduction in our communities.

## Methods

### Design

An interpretative phenomenological study was conducted with family members of individuals with lived experiences of mental illnesses using in-depth interviews with the support of a semi-structured interview guide. We pursued this study to unearth the essence, interpret, and describe the meaning of the phenomenon (familial mental illness stigma) among this population in Ontario (London-Middlesex area), Canada. The phenomenological research method allows for a “systematic, explicit, self-critical, and intersubjective study of its subject matter, of lived experience” [51] (p11). This method was chosen for the study because an interpretive phenomenology allows researchers to delve into and interpret language, lived experience, and social relationships of participants as well as the meanings ascribed to those experiences [52].

The study design enabled the researchers to establish an interpretative phenomenological stance to gain insight into the phenomenon while appreciating the participants’ narratives [53]. Interpretive phenomenology is also best enacted through the creation of an enabling environment that allows both the investigator and research participants to co-construct the meaning of the phenomenon through interactional processes including dialogue [51, 54, 55]. Ideally, interpretive phenomenology often moves beyond the recognition of the fundamental nature or outcome of the phenomenon (description) to unearth the meanings that humans ascribe to their lived experiences within a particular setting [52]. The interpretive phenomenology approach, together with interpretative phenomenology analysis, allowed the researchers and participants in this study the needed flexibility in the explanatory process of the phenomenon while the investigators observed the emergent themes as active members of the study as the interviews unfolded [56, 57]. That is, the interpretive phenomenology analysis is a subjective process whereby the researcher co-creates knowledge with those who have experiences to share, then layers on interpretation to this knowledge in a form of reconstruction [57].

The study was approved by the Western University Research Ethics Board **(#119602)**.

### Participants

Fifteen adults living in the Middlesex-London area in Ontario, Canada were enrolled in the study via criterion sampling which is suitable for phenomenological studies. By criterion sampling, we selected participants who met the predetermined criteria for the study which is their experience of knowledge or experiences of familial mental illness stigma. That is, we purposefully recruited persons who have lived experiences of the phenomenon under study and are willing to share their stories with the research team. These individuals include persons who are perpetrators of familial mental illness stigma and those who are not but have witnessed other family members stigmatizing their loved ones with mental illnesses. The participants were family members or close relatives of persons with mental illnesses. The demographic information of participants is presented in Table 1 in the findings section. All participants agreed to partake in the study by signing an informed written consent before the commencement of interviews.

**Table 1:** Participants’ demographic information.

Participant ID	Age Range	Gender	Relationship to a person with mental illness	FM Relative’s Diagnosis	Marital Status	Level of Education	Race/Ethnicity
FM_1	45–49	Female	Daughter	PTSD, Bipolar Schizophrenia,	Separated	Graduate	Caucasian
FM_3	55–59	Female	Sister	Schizophrenia	Married	Undergraduate	Caucasian
FM_5	25–29	Female	Sister	Substance use disorder	Single	Graduate	Caucasian
FM_6	50–54	Female	Sister	Schizophrenia	Married	Graduate	Caucasian
FM_11	50–54	Female	Daughter	Schizophrenia	Married	Graduate	Caucasian
FM_13	40–44	Female	Daughter	Postpartum depression/ Bipolar disorder	Common law partner	Graduate	Caucasian
FM_14	35–39	Female	Daughter	Depression/ Anxiety disorders	Common law partner	High school	White Canadian
FM_15	25–29	Female	Sister	General anxiety disorder	Single	Undergraduate	Middle East
FM_18	30–34	Female	Sister	Bipolar disorders Depression/ Anxiety disorders	Married	Undergraduate	White Canadian
FM_19	70–74	Female	Mother	Eating/Anxiety disorders	Divorce	Graduate	Caucasian
FM_21	40–44	Female	Daughter	PTSD	Married	Undergraduate	Caucasian
FM_24	30–34	Non-binary	‘Relative’	Depression/ Anxiety disorders	Single	Graduate	White European
FM_25	50–54	Female	Sister	Depression/ Borderline personality disorder	Married	Graduate	Caucasian
FM_26	60–64	Female	Sister	Schizophrenia	Common law partner	Graduate	Caucasian
FM_30	45–49	Female	Sister	Schizophrenia	Separated	College	Caucasian

**Key:** FM-Family members

#### Recruitment

The research team placed advertisements in family health practices, community health centers, and educational institutions to canvass participants. The advertisement was also shared via flyers on various social media platforms including Facebook, Twitter, and LinkedIn.

#### Inclusion/Exclusion criteria

Participant selection was based on the following criteria: (1) being 24 years or older; (2) self-identified as a family member or close relative of a person or persons with mental illnesses; (3) spoke and understood English; (4) being a current resident in London-Middlesex area; and (6) willingness to participate in the study for 45–60 minutes. A family member meets the inclusion criteria if they live in a separate residence or location from their relatives with mental illnesses within the study settings. The family member, however, should have some closeness with their relative with mental illnesses. We excluded all persons who identified as family members or close relatives of people with mental illnesses and were not resident in the London-Middlesex area to avoid anticipated variations in the availability of services and supports.

### Data collection

Data collection commenced on May 28, 2022, and ended on July 30, 2022. Participants were interviewed using a semi-structured interview guide. The interviews were recorded on Zoom and transcribed verbatim. Each participant was assigned a pseudonym by the interviewer to ensure confidentiality. Participants’ identities were further protected by ensuring that all voice files and transcripts were encoded and stored on a computer protected via encryption. The interview guide included questions related to participants’ experiences, and their understanding of familial mental illness stigma (see Table 2 for sample questions from the interview guide). Each in-depth interview lasted between 45 and 60 minutes. Participants were given $20 in appreciation for their time. All interviews were conducted by the first author of the manuscript, who is a doctoral candidate with two masters’ degrees and a registered nurse by profession.

**Table 2:** Interview guide.

1. So, before we get into specifics, I want you to talk about mental illness stigma more generally. What kind of mental illness stigma do you think people experience in Canada today?
2. Do you think stigma has gotten any better in recent years?
3. What do you think causes mental illness stigma?
4. You’ve been invited to this study because of being a family member of a person diagnosed with a mental illness. Can you tell me a bit about your family member who lives with a mental illness? a. How long have they been living with a diagnosis? b. What kind of support do they receive? c. How often do you see them? 5. How would you describe your experiences of having a family member who lives with a mental illness? a. What have been the most important things you have learned through this? b. What have been the largest challenges they have faced? c. What have been the largest challenges for you, personally? 6. Has this experience changed how you understand mental illness? a. [If yes] How so?
7. Now let’s talk specifically about stigma. Do you believe your [family member] experiences of stigma are because of their illness? a. What kinds of stigma do they experience? b. Have you ever been present when you saw them being discriminated against because of their illness? What was that like? 8. In this study we hypothesize that family members themselves might stigmatize their loved ones with a mental illness. Personally, do you feel like anyone in your family has acted in ways that are discriminatory towards your loved one with a mental illness? (a) How supportive have your family members been towards the care of your relative through their illness? b. [If you provide a lot of direct support yourselves] What kind of activities do your family members help you with? c. How do you feel about the help they give you? 9. Thinking honestly about your experiences through this all, do you think your attitude towards your loved one has changed since they started experiencing symptoms of mental illnesses? a. How was your relationship with your relative before their diagnosis? b. How close are the two of you now?
10. If you were ever to notice family members treating your loved one poorly because of their mental illness, how do you think you would address these negative behaviours? a. Is [family member] involved in family decision-making? If so, how often do you seek their opinion on important matters that need attention from all family members? 11. What is your opinion about other extended family and friends knowing about the diagnosis of your relative? a. Do you ever [yourself] feel embarrassed about their illness? 12. Has your relative’s diagnosis had any impact on you in terms of your relationships with others? How comfortable are you discussing your relative’s diagnosis with close friends? 13. In conclusion, what do you think can be done to reduce negative behaviours towards people with mental illnesses? 14. Do you have any questions or comments for me? 15. Demographic data: Age, gender, race and ethnicity, marital status, and level of education

### Data analysis

Braun and Clarke [58] technique for thematic analysis was employed to organize the data for the study. The approach involves six stages: (a) familiarizing oneself with the data; (b) generating initial codes; (c) generating themes; (d) reviewing the themes; (e) defining and naming themes; and (f) producing the report. In keeping with the analytic techniques, two members of the research team listened to the audiotapes of all interviews repeatedly and transcribed them verbatim into word processor files. We confirmed the accuracy of the earliest transcriptions by replaying the audio recordings and double-checking with the transcripts. We continued to reflect on the data by listening to the audiotapes and re-reading the transcripts while reflecting on each participant at the time of the interview to appreciate and interpret the context in which they spoke. This allowed the two researchers to account for the full, detailed, and thick description of the data. Having familiarized ourselves with the data, we manually started the coding process, underpinned by the research objectives, by assigning meaningful labels to segments of each transcript pertinent to the study using words and short phrases. The various initial codes were prearranged and organized logically into higher-level categories for thematic linkages or nexuses which paved the way for theme development. The two coders sorted the codes and mapped the various patterns into probable themes while combining all key coded data extracts within the identified themes. Another member of the research team independently reviewed the initial patterns established by the two coders. The three researchers then re-read the categorized data and recognized the commonalities and connections within the data to form the subthemes. All five co-authors reviewed and commented on the potential subthemes and themes to ensure both the relevance of the findings to the research questions as well as maintaining and uniquely representing participants’ voices or opinions. The analytic report was drafted by two team members and the full research team read and made changes to ensure succinct logical accounts of the data supported by substantial evidence and using direct quotes. In addition to the post-analysis interactions by the research team to establish the validity of the findings, five participants accepted our invitation to review and comment on the final themes via email. The participants then emailed their comments which were taken into consideration for the final interpretation.

## Findings

Themes were organized inductively from the study data to understand the lived experiences of familial mental illness stigma and how it can be reduced among family members. The participants’ views are articulated through five themes generated in our analysis. These included: (1) layered perspectives of social and family stigma; (2) family-related stigma; (3) complex interplay of family relationships and mental illness; (4) confronting stigma personally; and (5) envisioning a better future.

### Layered perspectives of social and family stigma

The layered perspectives of social and family stigma refer to the complexity of public stigma and its interconnectedness with associative stigma which has the drive to produce familial mental illness stigma. Participants’ viewpoints of their lived experiences demonstrate that they acknowledge the existence of such intricate connections between social and associative stigma. Familial mental illness stigma appears to have a link with social stigma. Familial mental illness stigma could exist when social stigma is at work within a social interaction. Family members often try to avoid social stigma, but in the end, they may perpetrate familial mental illness stigma which could further result in self-stigma of the affected person. Layered perspectives of social and family stigma comprised three subthemes: public/social stigma; social exclusion; and labeling.

#### Public/Social stigma

The World Health Organization (WHO) has explained public or social stigma in the context of health as a bias against people who may live with the diagnoses or symptoms of certain illnesses, and are treated negatively alongside their caregivers, family, and friends in every fabric of society [59]. Persons with mental illnesses are known to be socially stigmatized within their communities. Participants in this study reiterated the existence of social stigma in Canada against persons with mental illnesses. They noted the avoidance and /or discrimination of such people from certain employment opportunities as well as from performing certain key roles within society, which has further contributed to widening the inequality gaps in society. A participant expressed:

I think general ostracization, they are not included socially. It’s really hard for people with mental illness to get jobs and find housing in Canada. Sometimes it’s hard for persons with mental illnesses to get proper medical care (Participant 14).

Participants also contended that the ongoing stereotypes of persons with a diagnosis of mental illnesses are often extended to their family members. A participant recounted their ordeal with social stigma while caring for the younger brother living with schizophrenia:

“But with us, people just like wanting to stay away like nobody wanted to touch…including our extended family members. My brother himself was so ashamed as he lost some family members as they do not ask of him during family events” (Participant 6).

#### Social exclusion

Social exclusion in this study denotes an individual with mental illness’s inability to participate fully in activities within their family due to ongoing discriminatory behavior from relatives. This social isolation made affected persons feel separated from their loved ones. Some participants reported the difficulties associated with their family members being diagnosed with mental illnesses about interactions with their in-laws. A critical observation by a participant shows that their in-laws did not want to engage willingly with the brother living with mental illnesses compared with other members of the family:

My in-laws sometimes struggle to communicate with my brother diagnosed with mental illness. I sense the feeling of almost holding back from really getting to know him, and it feels like a very forced conversation when they’re talking to him, so … (Participant 18).

There is a lot of discrimination in the family, and I don’t agree with them. But just that he’s not a productive person so society owes him nothing. What’s the point of having him around the family I guess is the core idea (Participant 5)

Analysis of our data also revealed that some participants cut links with family members diagnosed with mental illnesses to save themselves from the psychological anguish often experienced by family caregivers. Other close relatives excluded affected family members to avoid the many hurtful reports about their relatives by others. One participant stated:

They [family members] have been hands-off. …, hurtful things have been said about my mom since her diagnosis. My sister said she did not want to be told how many times mom had been saved by laying on train tracks. I think she couldn’t allow that trauma constantly to disturb her. It’s too much. To maintain her own sanity and stability so that she is not in a constant state of panic and worry, you have to let her go, hence my sister deserted mom (Participant 1).

#### Labeling

There appears to be a relationship between labeling and internalized stigma which has the tendency to derail affected persons’ self-esteem and self-worth. Labeling within one’s immediate environment by family members could lead to self-stigma and loss of status within the family system. For instance, one participant narrated how their mom was labeled by their immediate family members because of her behaviours at social events. The presence of mom caused several members of the extended family to lose interest in some occasions:

My mom was seen as troublesome. …. During family get-togethers, Christmas, or any other occasion people wouldn’t come; many will immediately ask if mom is going to be there. And if she was, no one else was going to attend. Also, my ex-partner saw mom to be very difficult or dominant and would not like her around any time my mother-in-law visited us (Participant 1).

The labeling of persons due to a mental illness within their family could lead to a lack of social support and low self-esteem as well as loneliness within the immediate environment. A family member described how their mom labeled her son and kept calling him names due to a diagnosis of mental illness:

My mom has always labeled my brother as sick because of his mental illness diagnosis. She has always used that term, he’s sick. He has an illness, and it’s a very deficit,… And it took me a long time to realize that and to sort of try and counter that a little bit, and only in my later years have I been able to do that (Participant 11).

### Family-related stigma

Familial stigma herein is simply the extension of stigma to other family members by their relative’s experience. In this study, the fear of stigma from the public due to their relationship with the family member (associative stigma) resulted in some families excluding their relatives with mental illnesses from social events or socially distancing themselves. In hindsight, a participant stated vividly their discriminatory behaviors toward their brother due to a diagnosis. They failed to invite them to their wedding reception for fear of causing a scene during the celebration, which could lead to a loss of social status within the family. The brother’s exclusion from the wedding could also impact his mental health and validate any related self-stigma:

Yes, I have discriminated against my brother because I excluded him from my wedding. …, I got married at 25 years, and I didn’t invite him because he wasn’t mentally stable. I didn’t want him to cause a scene or require any energy that I didn’t have or didn’t want others to be distracted. I didn’t want him to ruin my perfect day (Participant 11).

A participant expressed their views concerning familial mental stigma using the following statements: “Yeah, my brother experienced stigma within his nuclear family since the whole family did not want anything to do with him because he uses substances. And he was subsequently sent out of the house by our parents” (Participant 5). While some participants demonstrated close relationships with family members living with mental illnesses, their children discriminated against affected persons by refusing to have dinner together:

In terms of discrimination, I think sometimes maybe my kids at 22 and 25; are not so enthusiastic about having a family dinner with their uncle. I think they might be if he did not have mental illness right because there’s not a lot of talk about and just his appearance going on…. that’s funny (Participant 3).

Related to family-related stigma is social distancing. Socially distancing in this context implies family members or close relatives of persons living with mental illnesses avoiding them and not wanting to have any close association with them, especially within the social realm. Living with a mental illness without a good family network could be impairing and ultimately delay recovery. Participants reported that some families avoided their loved ones with mental illnesses and dissociated themselves after their diagnoses instead of embracing and supporting them toward recovery.

He’s pretty much seen as not valuable or contributing to our family. We just don’t need to hang out with people like that I was always told growing up. But in our family, it’s a big thing like you don’t come around if you’re high and then don’t come around at all, because you aren’t working (Participant 5).

Another family member stated: “And other young people didn’t want to be around him. My brother died without any empathy from his friends” (Participant 6).

### Complex interplay of family relationships and mental illness

The relational interactions families have around mental illnesses sit within the context of already frequently complex familial relationships. Family members subjectively perceived that complex relational experiences are intertwined with their perspectives on mental illnesses. These subjective experiences of family members were discussed under the following subthemes: loss of social status within the family; exclusion from family decision making; divorce/loss of romantic partners; neglect due to difficult behaviors; and compassion.

### Loss of social status within the family

Several participants felt their loved ones with mental illnesses were deserted by family members at a time when they needed affection and support from them to improve their mental health. They described how some close relatives with mental illnesses were devalued leading to the loss of their social status within their family following a mental illness diagnosis. Some of the family members did not even consider their sick relatives as part of the family anymore:

Yes, within our extended family, only a minority of people would ever ask about my brother because of his mental illness diagnosis. Otherwise, he was just not brought up or he was not mentioned, and if you mentioned him, people would just try to change the subject. So, they never offered any help being it emotional or practical until he died (Participant 6).

Positive relationships, together with good social support networks within one’s family are well-documented for mental health recovery. However, several participants in this study reported that their loved ones’ mental illnesses brought conflicts within the family. The severity of their illness compelled some relatives to cut contact with the affected person. A participant described how their husband who was supportive of the relative’s care also complained several times about the in-law’s exaggerations:

Mental illness is ingrained in our family and most of my mom’s sisters have suffered from it. They’ve had periods of conflict and not spoken so… I’m sure my cousin doesn’t speak to my mother anymore. They have no close relationship though they were somewhat close in my younger years. My husband who supports me has said many times that my mom exaggerates. I think … we all move through ups and downs in life. But the chronicity of my mom’s sort of helplessness… (Participant 25).

#### Exclusion from family decision making

The exclusion of persons with mental illnesses from family decision making after a diagnosis could be tantamount to loss of status within one’s family, especially if the affected persons were consulted on important family matters before their diagnoses. Some study participants discussed how their family members were excluded from all family decision-making due to a diagnosis of substance use disorders, a move that can affect one’s self-esteem:

My brother is not involved in any decision-making in the family. He’s cut off from all family activities. Even his own mother’s care in the nursing home, the mother obviously just doesn’t prefer him to be involved. I suppose or trust that he can make those decisions, but he’s definitely not involved at all (Participant 5).

Several participants’ shared their views on the exclusion of persons with lived experiences of mental illnesses from family decision-making and indicated how these family actions can further inhibit affected persons from improving their mental health. A participant stated:

My brother is often involved in family discussions, not decisions. We had a death in my family a couple of years ago and he was involved in those discussions. …, just to make sure that everyone was on the same page and understanding but when it came down to decision-making, he was not involved in that process (Participant 18).

#### Divorce/Loss of romantic partners

If an individual has a romantic partner, their support can be key to managing health challenges, including mental illnesses. Nevertheless, some participants’ stories in this study indicated that their partners mistreated them while struggling with a mental illness. A family member mentioned:

Mom was divorced after 35 years of marriage and dad deserted her because of her mental illnesses. I assisted her by purchasing clothes, setting up things like a meal on wheels, essentially just talking to her every day, and later became her advocate and got her into community services until she went into in-patient care due to frequent relapses (Participant 1).

Another participant expressed this concerning divorce issues connected with family relationships and mental illnesses:

Mom was beautiful… and I had a very positive regard for her as a child. Mom was divorced by my dad due to her mental illness diagnosis. …, I started to have a different opinion of her when she was hospitalized (Participant 25).

#### Neglect due to difficult behaviors

This subtheme describes how persons with mental illnesses were deserted by some family members due to their atypical behaviors or inability to keep boundaries. A family member stated:

I was about 20 years old when I left home to start my own life. My brother started living on the street by the time I returned. I lost track of him for a little while and then found him in an apartment. I moved out West … and I didn’t have a lot of contact with him for several years. My mom told him to leave because they started having a lot of conflicts and just my mom as a single mom and my brother became very difficult, very delusional partying all night with drugs. He was not helping around the house, and he dropped out of school. My mom received advice from the therapist or somebody that she should kick him out of the house for her preservation (Participant 11).

The increased neglect of persons with mental illnesses by family members can further worsen their already weakened mental health due to a lack of social support and emotional stress. Participants’ accounts showed that such actions protect families from psychological stress and prevent family members from developing mental health problems themselves:

I was seeing my mom about once a week. There was about four years after her diagnosis when we did not have any contact and that was a time when I had two kids, and they were small, and mom’s behavior was dramatic, unpredictable, and demanding. And it was happening over some time like a couple of years where there was a growing concern. And we had an argument, and she was very mean to me, and rude to me verbally, and hung up on the phone on me, and I just decided that I needed to set a boundary for my own well-being, and I didn’t reach out to her, and it was very difficult. One of the hardest things I have gone through, yeah and then I decided to reconnect and wrote a letter, and we began to reconnect again (Participant 25).

Other participants disclosed how their loved ones’ difficult temperament and abusive nature caused them to stay apart:

I normally see my dad a few times every year. I haven’t seen him at all since the pandemic, but… Dad has anxiety and anger problems, so it is really hard to deal with him. And even though he did go for anger management, he still struggles with his anger. Dad was abusive in the past and he still sometimes does not agree with you (Participant 14).

#### Compassion

Whereas some participants in the study expressed disappointment regarding the challenging behaviors of family members with mental illnesses, others were compassionate towards their loved ones after diagnosis. Certainly, some participants at times got frustrated at the turn of events, but they remained committed to the care of their family members:

I don’t think my attitude toward my daughter has changed since her diagnosis. I’ve always been supportive and continue to do my best for her. I’m sure I get frustrated sometimes, but I don’t know if that’s really a change (Participant 19).

Another family member revealed:

…my attitude towards my brother has changed over the years since his diagnosis because it’s been so long. It’s been many decades and I’ve changed and grown as an adult. And he hasn’t gone through the same life phases and ‘normal’ life phases (Participant 11).

Participants’ accounts also pointed to compassion towards the affected person with mental illnesses within the family because family members show maturity and understanding of relatives’ mental illnesses. Some family members also engaged their loved ones in an open conversation to restore hopes and aspirations in life despite the diagnosis:

As I got older, and his mental health got more severe leading up to his bipolar diagnosis it changed completely. The biggest thing that changed for me was when he first attempted suicide. That was a huge awakening really for all of us because we always took him seriously but once that happened we became very alert. It was scary and so we realized that we needed to support him in any way that we could. … as I’ve gotten older, and I’ve had more open and honest conversations with him (Participant 18).

With respect to how compassionate some family members are to their relatives with mental illnesses, one participant remarked: “Her brother probably is more engaged with her than before. Because she does reach out to him more and I believe he understands that she needs the support” (Participant 19).

### Confronting stigma personally

Many participants shared ideas about how familial mental illness stigma could be confronted at the family level knowing that family members and close relatives can be the perpetrators of stigma. Participants discussed confronting stigma personally under the following subthemes: familial education on mental illness; initial concealment/strategic disclosure; selective versus full disclosure; non-discrimination; and advocacy against familial prejudice.

#### Familial education on mental illness

Some participants underscored the importance of family education on issues of mental illnesses in reducing familial mental illness stigma. These family member participants noted that in-depth knowledge of mental illnesses is the way forward to understanding the lived experiences of their loved ones with mental illnesses and appreciating them within the family. A participant indicated how this could be achieved through social contact-based education:

Individuals with mental illnesses deal with stigma by educating their family members and themselves. They find good support and ensure that they are confident in themselves. Stigma is a lack of education on mental illnesses… understand that mental illnesses are something we all experience, and we can accept it and help one another (Participant 15).

Several family members in this study proposed family education by persons with mental illnesses. This approach acknowledges the expertise of those living with mental illnesses in their own experiences and as educators of others. Awareness was seen as a pathway towards reduced stigma:

I think it is challenging for individuals diagnosed with mental illness to deal with stigma, including familial mental illness stigma. They first need to understand their illness and know that their illness is no different than cancer or diabetes. They need to understand their illness so they can explain it to others. I think they need to be opened to talking about their illness and perhaps find the people in their lives and family that are more understanding, empathic, and willing to support them. Not everyone in your circle or … but some family members will try to listen and try to understand them … I think the more awareness we have about mental illness and stigma in general the more we can change the stigma (Participant 21).

#### Initial concealment/ strategic disclosure and full disclosure

While stigma may be directly confronted, other approaches of avoidance were noted. Limited disclosure was one means of avoiding stigma. Most participants in our study revealed both selective and full disclosure of a mental health problem of their relatives to other family members or the public as a form of coping with their loved ones’ illnesses as well as the associative stigma at the family level. Selective disclosure implies that not all persons within their social space or family will be informed about the mental health problems of the affected family member. Participants’ views show that they used selective disclosure to reduce the stigma connected with mental illnesses. One of the participants said:

There are levels of privacy I would say depending on who I am interacting with and ways that I would deal with someone’s attitude towards my family. If it is a distant person at a social event, for example, I would just be very factual describing briefly that my brother lives with a chronic disability, I might say schizophrenia, but I might not depend on whether I want that person to judge me or not, or…I make an in-the-moment decision about whether the person is trustworthy, meaning whether they will think negatively of me, or… some base factual knowledge themselves (Participant 11).

Another participant recounted:

I am still not very comfortable talking with extended family members about my brother’s mental illness. But I think with friends, we may talk about it. That is, with really close friends. I don’t think that I would want to talk about it with anybody at my workplace. Colleagues may sometimes see something in the news like a mother losing a child or something, right, and they’ll make a comment, and I might need to remind them that my mother lost a child, and they don’t seem to equate it in the same way (Participant 6).

Some participants also adopted full disclosure as an approach to reducing mental illness stigma. Some participants were fully open about their loved ones’ mental health conditions by discussing them with friends and other family members. A family member disclosed how being open about their relative’s illness offered them the opportunity to reduce stress by sharing their problems with others, allowing for advice and support when need be:

I was kind of embarrassed three decades ago… anything about it, and I thought that he would recover at some point or get better. I’m pretty open about his diagnosis even though I don’t go talking about his diagnosis. I think it’s much better now than it was 30 years ago I can say to people I have one sibling, and …. And he’s not able to work and I’m his caregiver and I do not need to hide it anymore (Participant 3).

Some participants noted that talking about mental health conditions within families was an opportunity to educate other people on mental illnesses and psychotropic medications. The participants emphasized how beneficial disclosure was to their mental health as it relieved them of the pressure and psychological stress of having to conceal their family member’s condition from other relatives, close friends, and co-workers. A participant mentioned:

I’ve gotten a lot less shy about telling people that my brother had mental illness… you know I have a brother who lives with paranoid schizophrenia. He lives in a special housing situation for people who were on the street, and now he’s not, and when people who will often say, well, if they just take their medication… Then I pipe up and say you know that’s not the way… I will try to educate people to inform them that it’s not just about taking medication. Taking medication is not a complete solution for anybody or everybody who has mental health challenges. It’s more than that…. (Participant 26).

#### Non-discrimination

Some family member participants expressed how they developed expertise in understanding mental illnesses. Their understanding of mental illnesses led to the provision of social and practical support to affected family members without any form of prejudice. The absence of prejudice within the family environment brought inclusiveness among households which was good emotionally to improve their mental health. This valuing of non-discrimination was expressed by one of the participants:

Not that I know, I don’t think she experiences any stigma within the family. I think generally her perfect family seems to have an open mind and understanding of her condition. I think we’ve all become more informed throughout the process now compared to perhaps the early stages when we had some misconceptions about it. I know that my son has concerns about the medications, but I don’t know if there may be some stigma associated with that (Participant 19).

Some participants were more explicit in sharing their views on the open-minded or unbiased attitude of family members toward relatives with mental illnesses. It was noticeable that several participants were well-informed about their relatives’ diagnoses and agreed to support them on the path to recovery: “I don’t think anyone within our family will stigmatize her because of the diagnosis. As a family, we’ve been educated about her diagnosis, and everyone knows what to do to support her. We really understand the problem at hand” (Participant 15).

In the same vein, a participant narrated how their loved one was treated by the family post-diagnosis:

I would say nobody in our family stigmatizes my brother because mom was still alive when he was first diagnosed, and she spent quite a bit of time with him… My sister, a nurse practitioner, was very good about dealing with him. My other sister who suffered from mental illness herself was very open and took him to live with her for 10 years. I would… but he and I were closest growing up. We’ve continued to communicate, and I haven’t had as much direct contact with him. Our in-laws and children have been fine and comfortable with him. He came home for our mom’s funeral and people approached him and talked to him (Participant 26).

Other participants reported the need for the media to desist from negative reportage on mental illnesses and portray positive media stories to project the good things some persons living with mental health problems do to support their communities. They remarked:

I think changing what we see in media concerning persons with mental illnesses will help in reducing the ongoing stigma. I believe having buried normal boring people with mental illness more represented that it’s just an idea that if you have a mental illness, you can live a normal life and you aren’t crazy or evil, you’re just kind of normal. I think especially with the more serious diagnoses like schizophrenia; we have a fear that it’s somehow scary or bad when in fact it’s just people living their lives. I took a psychology course and realized how wrong I was about what I thought schizophrenia was until I was taught. That was just my perception from the media growing up about the idea that people with mental illnesses …. (Participant 24).

If I hear people saying things that are not true about people with mental illness, I will definitely speak up and tell them that what they’re saying is not valid. It is surprising to me today when people discriminate against people with mental illness because if you understand mental illness, you will realize that people would never choose that life intentionally. I guess just talking about it right in the media, healthcare settings, and health classes in high school about what mental illness looks like, people wouldn’t discriminate against persons with mental illnesses (Participant 3).

#### Advocacy against familial prejudice

Some participants discussed strategies they used in dealing with the unfair treatment of family members, whereas others suggested approaches they will employ in avoiding maltreatment of their loved ones should it arise. Many participants planned to intercede on behalf of their loved ones by calling out stigma and taking the opportunity to educate others on mental illnesses and the associated misconceptions. According to a participant, the involvement of family members in public education initiatives should be a way to bridge the ‘us and them’ gap within the public sphere:

If [I] found any family member abusing my mom because of her diagnosis, I would call them out on it. And I would try and educate them on what I know about depression and bipolar disorder as well as mental illnesses, in general, to help reduce the ‘us and them’ gap (Participant 13).

Similarly, another participant explained how they supported their family member against familial prejudice within the household:

…I called my sister who deserted Mom for two decades for us to deal with Mom’s belongings. After a long struggle, my sister did come with her husband and one of their sons, at 19, who had never seen Mom before…they all had something negative to say about Mom. I quickly jumped in and defended Mom by telling them that Mom had been in psychosis for the last two years, so her home was in disarray. Please, as you enter the apartment all things that come out of your mouth need to have a lens of empathy. As mom was sick and didn’t have the needed support hence, I had to address this very firmly with my sister. That is, I didn’t want to hear snide remarks that she was not part of the last 20 years of, you know, being a part of her life…. (Participant 1).

### Envisioning a better future

While narrating their lived experiences with mental illnesses over the years in the care of impacted relatives, family members noted that transforming services would reduce the social harms that may be translating into stigmatizing actions of family members. The resulting themes included: reforms in access policies to mental healthcare and free access to quality support services. These suggestions by family members, however, were contingent on adequate funding for quality mental health programs at all levels to support persons with mental illnesses.

#### Reforms in access policies to mental healthcare

Transformations in access to mental healthcare included recommendations that policy-makers should ensure the availability of care to all who need it. Participants considered free access to care as a key step to improving the well-being of individuals with mental illnesses and the de-stigmatization process, which could lead to better health and social outcomes. For instance, a participant stated:

I think policymakers/governments could do a better job. There is the need to do more public education around it [mental illnesses]. In terms of policies, I think my daughter has received very good care and it’s never been a case where she hasn’t been able to access it. She can have access to the crisis team, which is working for her… lots of people may be having challenges with access hence policy-makers could improve access to care for people with mental illnesses (Participant 19).

Another participant reflected on the lack of diversity of available support services for persons with mental illnesses with respect to both older and younger populations, which to them, tends to benefit the younger generation with more interventions. The participants believed there should be more outreach and less confined ways to ensure diversity in terms of who can access service:

…there’s an older generation of individuals like my brother, who have been living with a diagnosis for so long and they’re caught between worlds. People who are more newly diagnosed are getting the benefit of the changing approaches to care which are becoming more compassionate and becoming more trauma and violence-informed. But people like my brother have gone too far into being discriminated against and mistreated and excluded to the point where there are no real programs that can help them. …. (Participant 11).

…, Informing people about the full spectrum of what to expect during medication treatment and how to come off is necessary. I think we need to create a world that makes space for people with mental illnesses. I believe we need more expanded ways of including people in our world that live differently. There should be more counseling access to replace medication or psychotropic drugs (Participant 25).

#### Free access to quality support services

The provision of adequate and free mental health support services in our communities was advocated by several participants. During the interviews, participants advocated for more creativity in mental health-related system design as well as adequate funding for mental health programs. One participant explained:

…. challenging to figure out how to deliver services. Better and more person-centered, but I think that there’s room for more creativity in how those policies roll down the programs they run. And I think we need more funding for people’s mental well-being. I think people need access to key publicly paid-for short and brief interventions (Participant 25).

Likewise, other participants proposed: “I think policy-makers can do more to support persons living with mental illnesses in our communities. There should be more free quality support that’s accessible” (Participant 24). Another participant intimated:

I believe that policy-makers should do more to help people with mental illnesses who are struggling with a disability. Checks are very limited, so if my brother didn’t have a house that my mother bought him, he would have been in the worst condition by now (Participant 30).

Other participants bemoaned the current mental health system in Canada and demanded a reorganization of mental health services to pave the way for short wait times for psychological assessments for persons with mental illnesses for possible diagnosis and treatment. Participants felt this initiative could incorporate both public education on mental illnesses and available support services to tackle the stigma of mental illness at all levels of society:

… I think is vital to have a proper foundation of mental healthcare. I think a lot of the time when you go into emergency departments, the mental health capacities are just overflowing with people who are requiring assistance. ……proper support is essential in our healthcare system to reduce the wait times for some of those programs, especially for initial assessments is just heartbreaking… (Participant 18).

## Discussion

We conducted this study to explore the lived experiences of family members of persons with a diagnosis of mental illness and how they enact or address potential familial mental illness stigma. The findings of this research revealed both the potential for the existence of familial mental illness stigma at the family level perpetrated by some family members or close relatives towards their loved ones, along with actions taken to prevent or reduce this stigma. It is worth noting that the high educational levels of participants might have influenced their perspectives of familial mental illness stigma as high education can relate to high income, and therefore access to additional private supports. As well, the gendered nature of the data could potentially influence participants’ views on familial mental illness stigma as the data for this study primarily involved the views of women, with only one gender-diverse person. Therefore, our findings missed out on important cultural nuances of different families and different perceptions of mental illness as stigma experiences may differ across different ethnic and cultural groups within Canada since all participants were White Caucasians except one person who identifies as a racialized person from the Middle East. Participants’ stories showed that familial mental illness stigma is interconnected with other social sources of mental illness stigma. Blame was also pointed towards healthcare systems wherein inadequate services were rebuked for increasing the types of stressors that might lead to discriminatory behaviours. The more individuals were forced to depend on their immediate families for daily needs, the more tension was felt within some families. In the end, participants of this study underscored potential policies and interventions necessary to transform the existing services to make persons with mental illnesses more independent. Our study adds to the existing body of literature on various forms of stigma, filling in some of the gaps, specifically how family members themselves navigate familial mental illness stigma [33, 35, 60–64].

Stigma within families does not sit apart from other family dynamics, but rather the multi-layered interconnectivity of family relations pre-dates diagnosis and the accompanying symptoms of mental illness. These complexities sometimes add to both positive and negative existing familial relationships. For instance, some families responded to symptoms and diagnoses with compassion, and in most cases, family members provided resources to support the mental health of their relatives; however, it was noted that at times this support was focused on family members protecting themselves from associative mental illness stigma. Where these concerns were the greatest, some family members isolated their loved ones living with mental illnesses. This might have unintended harms related to the prevention of access to support. These findings are analogous to those on the fear of associative stigma that compels some family members to reduce social contact with their relatives with mental health issues and in the end, stop supporting them both emotionally and practically [33, 64–66]. Some family members engaged in concealment, which may have had negative implications for access to care. This social exclusion was also extended to other social events within their local communities. These observations align with other findings that most persons with lived experiences of mental illnesses are excluded to some degree from family and community participation [32, 33, 60, 67]. Such exclusions have implications on the self-worth and personal empowerment of the affected individual, which in turn, could prolong their recovery.

The study participants acknowledged frustrations and stressors connected to the care of persons with mental illnesses as part of the complex interplay within family systems and situated blame for mental illness stigma within this experience. The frustrations included blaming the individual experiencing an illness for the symptoms of their condition. This reproach is felt by the individual and can become a barrier to recovery as they hide their challenges from family members. In this context, disclosure is challenging, often ‘a double-edged sword’ as those living with mental illnesses navigate the potentials of either support or stigma from their loved ones [68, 69].

Our findings revealed that if family members lack knowledge or carry societal misconceptions about mental illnesses, they can perpetuate social isolation and emotional distress of their loved ones. Our participants highlighted other family members who would exclude those with mental illnesses from opportunities to exercise autonomy or power within the family unit. This exclusionary approach parallels experiences that persons living with mental illnesses encounter devaluing stereotypes in other social contexts such as school or workplaces [22, 67, 70]. Therefore, there is no guarantee that all those who face exclusion in broader society will find solace within the family unit.

While participants noted the ways that their loved ones are stigmatized within their families, they also provided recommendations for how society can do a better job of creating a supportive context. Some of them focused on individual-level interventions such as increasing understanding of mental illnesses within families. The family members also suggested transformative education with a focus on social contact-based education to correct some common fallacies and fears about mental illnesses. This is grounded in the idea that erroneous beliefs regarding mental health issues have contributed to the persistence of public stigma as well as stigma within families. Using positive media stories and social contact-based education with intentionality is promoted, where persons with lived experiences are given platforms to exchange ideas with members of the public on mental illnesses to reinforce societal understanding of mental health problems. This approach is evidence-based and tends to reduce the rising social stigma in public spaces which could have a ripple effect on other forms of stigma [65, 66, 71, 72]. Participants noted that addressing the broader social stigma of mental illnesses should translate positively into family interactions as well.

A focus on recommendations for improving understanding was related to the ideas of diagnosis, familial education, and disclosure. Participants’ reports on strategic disclosure or selective versus full disclosure are consistent with previous studies, which revealed that strategic disclosure allows individuals to find the safest ways to tell their stories [35, 60, 73–75]. If individuals have the luxury to educate their family members before full disclosure is made, it can create a safer context [6, 62, 75, 76], including the prevention of familial mental illness stigma. Family members’ understanding of their loved one’s mental health challenges through familial education by the affected individuals could potentially lead to both practical and emotional support within their immediate environment.

### Implications for practice

Health care professional’s ability to advise family members on their potentially stigmatizing beliefs during service delivery is critical to reducing the ongoing familial mental illness stigma. Clinical practices should focus on family-centered care where persons with mental illnesses, family members, and service providers can play vital roles in advocating for the health and well-being of the affected individual. By this approach, service providers may be able to uncover any hidden distress within the family connected to their clients’ illnesses. Health providers can then advise them based on their strengths and weaknesses and the voices of lived expertise should be included in the best approach for stigma reduction. As educators, health professionals can play a role in supporting high-quality public messaging about mental illnesses more generally, as well as targeted stigma reduction.

### Directions for future research

Our study revealed that familial mental illness stigma is ingrained in the broader societal stigma. The reduction of familial mental illness stigma partially depends on a decrease in broader social mental illness stigma. Further research is needed to critically explore the connections between familial, public, and associative stigma to ascertain a baseline to inform anti-stigma programs seeking to reduce familial mental illness stigma in high-income countries and beyond. Researchers in high-income countries should undertake longitudinal studies that include persons with mental illnesses, family members, and health professionals to investigate the relationships between stigma by association and familial mental illness stigma to unpack potential intervention points both within and beyond the family system. Also, researchers should plan to combine the knowledge of mental health professionals, individuals with lived experiences of mental illnesses, and family members in more intervention-based work that transforms stigma and enhances recovery experiences.

### Policy implications

While the aforesaid approaches look to reducing stigma at the individual level, participants highlighted that the transformation of healthcare systems would create a better context for stigma to be mitigated. Unfortunately, the social stigma of mental illnesses continues to be present in the fabric of society, including across high-income countries. Reducing social stigma is an ambitious but necessary endeavor to support families and their relatives with mental illnesses.

Further, participants in this study identified the need for health systems reforms where equal attention will be given to curative and preventative services. Participants felt reforms in the mental health systems, particularly access and availability to care could be fundamental to reducing the wait time for initial assessments to avoid undue complications before a diagnosis is determined. Early detection and treatment have been found to result in a good prognosis. Good policies with adequate funding for programs to support persons with mental illnesses in the management of their symptoms will be vital in bridging the existing ‘us and them’ gap within the social realm.

### Limitations

Our findings represent the perspectives of family members (particularly women) of persons with mental illnesses in the Middlesex-London area in Ontario, Canada. Data were collected through the Zoom platform due to the Covid-19 restrictions which made it difficult to do any direct recruitment of participants to increase diversity of experiences. Therefore, there are potential limits to the transferability of these findings to other social contexts. Also, since the researchers sought to recruit only persons who could speak and understand English, there are limitations related to diverse cultural experiences of stigma, a culturally mediated phenomenon.

### Conclusion

Our findings confirm the existence of familial mental illness stigma from the viewpoints of family members of persons with mental illnesses in a high-income country and add to the growing body of literature on mental illness stigma. While the presence of familial mental illness stigma is acknowledged in current literature, the particularities of how this is lived and navigated have received little study. Family members largely situated familial mental illness stigma within broader stressors of limited services as well as broader social stigma. Confronting stigma both directly and at these root causes should improve the well-being of those living with mental illnesses, including openness of access to services. Participants suggested the need for more social contact-based education and positive media messaging to correct ongoing misconceptions about mental illnesses. The study participants also recommended reforms in health service access and community services to better equip individuals with lived experiences of mental illnesses to meet their needs and ultimately optimize empowerment and independence. Therefore, the transformation of familial mental illness stigma sits within other calls for enhancing mental health and social services across Canada.
