# Correction: Negotiating familial mental illness stigma: The role of family members of persons living with mental illnesses

**DOI:** 10.1371/journal.pone.0333321

**Published:** 2025-09-29

**Authors:** Joseph Adu, Abram Oudshoorn, Kelly Anderson, Carrie Anne Marshall, Heather Stuart

After publication of this article [1], the corresponding author noted that errors were made in the presentation of the participant quotes in [1]. Specifically, the published quotes in [1] are not the raw study data (interview transcripts) and instead represent abbreviated quotes used during analysis as succinct logical accounts of the data collected. The correct participant quotes which represent direct quotes from the raw study data are as follows:

- Participant 14: ‘Um, I [participant] think definitely general ostracization not included socially. I also think it’s really hard for people with mental health issues to get jobs and find housing. And sometimes it’s hard for them to get proper medical care as well because sometimes if someone has a mental health issue. A doctor focuses on that and doesn’t take their physical health, as seriously...’
- Participant 6: ‘…, but with us, people just like wanting to stay away like nobody wanted to touch that. My brother himself was so ashamed you know and not just him you know like I remember one time when he was released from hospital, and his roommate, um, you know came in and would visit with us all the time because he said like he had lost all his friends and his family didn’t want to be around him, because he had been in the psychiatric hospital…’
- Participant 18: ‘… Um, they [participant’s in-laws] sometimes struggle communicating with him [participant’s brother]. … for me, I sense the feeling of almost like holding back from really getting to know him, and it’s just, it feels like a very forced conversation when they’re talking to him. …’
- Participant 5: ‘… I think [there] is a lot of discrimination [in the family], and I don’t agree with. But yeah, just so, he’s not a productive person, so society owes him nothing, and he doesn’t contribute. So what’s the point of like having him around, I guess is the core idea?...’
- Participant 1: ‘… They [family members] have been hands off. … great many mean and hurtful things have been said. For example, …, my sister said I don’t want to be told how many times she’s been saved through laying on train tracks. I don’t want to know. I think for her [participant’s sister] she couldn’t allow that trauma constantly. It’s too much. So, in order to maintain your own sanity and stability so that you are not in a constant state of panic and worry, you have to let her go …’
- Participant 1: ‘Yes, oftentimes when it was the family get-together, say Christmas or any other [occasion], people wouldn’t come. They [family members] immediately ask … is your mother going to be there? And if she was, no one else was going to attend. That’s a clear… and my ex-partner, when he had his mum, we would try to have both mums together, and of course, under normal circumstances having two mothers-in-law together in one place is often tenuous. Too very dominant… but they would never sit in the same [table] together’
- Participant 11: ‘… My mom has always labeled him [my brother] as sick. … she [mom] has always used that term. He’s sick, he has an illness he’s sick, and it’s a very deficit, …. And it took me [participant] a long time to realize that, and to sort of try and counter that a little bit, and only in my [participant] later years have I been able to do that …’
- Participant 11: ‘… Yeah, I [participant] excluded him [my brother], I didn’t invite him to my wedding. I was just thinking about this the other day. Uh, I got married when I was … years, and I didn’t invite him because he [my brother] wasn’t mentally stable. I didn’t want him to cause a scene or require any energy that I didn’t have or didn’t want others to be distracted. I didn’t want him to ruin my perfect day, …’
- Participant 5: ‘…Oh, yeah, definitely like our whole family doesn’t want anything to do with him because he uses substances. …I [participant] have this brother who has substance use that’s kind of like shunned out from our family, and considered what my parents would say a low life and disowned because of his substance use …’
- Participant 3: ‘In terms of discrimination, I [participant] think sometimes, so my kids are 22 and 25. And I’ll say, let’s have a family dinner with your uncle, and I think they’re not as enthusiastic about it as they might be if he did not have [a] mental illness, right, because there’s not a lot to talk about. And just his appearance going on…that’s funny’
- Participant 5: ‘… He’s [participant’s brother] pretty much seen as not valuable or contributing to our family. We just don’t need to hang out with people like that, I [participant] always thought like growing up. I felt like you should still include people anyway. … But yeah, in our family it’s a big thing like don’t come around if you’re high and then don’t come around at all, because you aren’t working…’
- Participant 6: ‘…And other young people didn’t want to be around him [participant’s brother].… And then, when he died, it was interesting to me who came to the funeral and who did it….’
- Participant 6: ‘… A minority of people in the extended family who would ever actually ask about him [participant’s brother]. Otherwise, he [participant brother] was just not brought up; he was not mentioned, and if you mentioned him, people would just try to change the subject…’
- Participant 25: ‘… It’s [mental illness] so ingrained in our family so at times they have not spoken. They’ve had periods of conflict and not spoken so whether that’s my aunt, discriminating [against] my mom, or setting up a boundary… I’m not sure if my cousin doesn’t speak to my mother anymore. They have no close relationship [now], they were somewhat close in my younger years. But now my cousin tells me you know she doesn’t have time for my mom…. And then my own family, like even my husband [who] supports me … I know has said many times, like you know your mom exaggerates,… But the chronicity of my mom’s sort of helplessness …’
- Participant 5: ‘… He’s [participant’s brother] not involved at all [in family decision making]. He’s really cut off. He’s not involved, for example, his mom, who’s also my sister’s mom …is in a nursing home and she made my sister a power of attorney for decision-making as well as her partner so he’s not involved in any of that even in his own mother’s care because his mother obviously just doesn’t prefer him to be involved. I suppose, or trust that he can make those decisions [but] he’s definitely not involved at all. …’
- Participant 18: ‘He [participant’s brother] would be in discussion. So, we had a death and my family a couple of years ago, and he was involved in those discussions. For sure, just to make sure that everyone was on the same page and understanding, but when it came down to decision-making. He was not involved in that process…’
- Participant 1: ‘…, she’s [participant’s mom] divorced, so her marriage of … years ended in... My [participant] dad finally had enough, the paperwork went through and the divorce was finalized. He [participant’s dad] was out of the picture, so she didn’t have a partner. My [participant] younger sister had to draw some boundaries; she hasn’t spoken to my mother in 20 years, and she lives here in [the city]. She’s had children that my mother has never met. So my sister had built a very large wall. She pretty much disowned her [mom]…I chose to be the person who would be of assistance to her…’
- Participant 25: ‘…She [mom] was beautiful and] I had a very positive regard about her as a child. …She started to change in her life, and she being a parent, wasn’t her main focus anymore. She became divorced and she… party[ing], and that became her focus…. …. I started to have a different opinion of her … when she was hospitalized…. with experience of going through withdrawal from taking a cocktail of different pills…’
- Participant 11: ‘I [participant] left home when I was about 20 [years]. …He [participant’s brother] was living on the street at that time. So I would just sort of see him sometimes on the bus, and I’d see him on the street. I lost track of them for a little while, and then I found him in an apartment. … He was really sick, he’s really delusional and like super skinny, and help to get them back into the hospital to get some help. My mom told him to leave. …they were starting to have a lot of conflict, and the conflict. My mom [was] a single mom, and my brother became very difficult [and] very delusional… He was staying up all night and using drugs and partying and not helping around the house …’
- Participant 25: ‘I [participant] see her [mom] about once a week. There was a period of time about 4 years, where we did not have any contact, and that was a time when I had 2 kids, and they were small, and her behavior was dramatic and unpredictable, and demanding. And it was happening over a period of time, like a couple of years, where I was growing more concerned. And we had an argument, and she [mom] was very mean to me, and rude to me verbally, and hung up on the phone on me. I just made a decision that I needed to set a boundary for my own well-being, and I didn’t reach out to her, and it was very difficult. One of the hardest things I’ve gone through, and then I made the decision to reconnect [through] a letter, and we began to reconnect again.’
- Participant 14: ‘…I [participant] normally would [see dad] a few times a year. I haven’t seen him [participant’s dad] at all during the pandemic, but we have kept in touch a bit. I would say on the front line because the way his anxiety manifests is usually anger. That is really hard to deal with. And even though he did go to anger management, he still struggles with his anger. So, … he was abusive in the past, and he still sometimes…. But I am not saying that’s because of his mental health, but it’s a behavior that he has’
- Participant 19: ‘I [participant] don’t think so [my attitude toward my daughter has not changed since her diagnosis]. I think I’ve always been supportive and so I think that has continued. I’m sure I get frustrated at times, but I don’t know if that’s really been a change…’
- Participant 11: ‘Yes…it’s been many decades, and I’ve changed and grown as an adult. And he [participant brother] hasn’t gone through the same life phases and ‘normal’ life phases …’
- Participant 18: ‘But as I [participant] got older, as his [participant’s brother] mental health got more severe and leading up to his … diagnosis, it definitely changed. The biggest thing that changed for me was when he first attempted suicide; that was a huge awakening, really for all of us, because we always took him seriously, but once that happened, it was scary, and so we realized that we needed to support him in any way that we could... So it’s definitely changed as I’ve gotten older, and as I’ve had more open and honest conversations with him as well’
- Participant 19: ‘Well, I [participant] think maybe my son, her brother… probably is more engaged with her. Because I think she does reach out to more [to him], and I think he understands that she needs the support’
- Participant 15: ‘I [participant] think we need to educate. I think that we need to let people know what mental illness actually is, that it’s not a one-size-fits-all. … if you’re faced with somebody with a mental illness, how would it be supportive, because without education, we can’t decrease the existing stigma, …’
- Participant 21: ‘I [participant] think it’s hard for a family to support someone with a mental illness… and some family members are better equipped to deal with it than others. … I think there’s a lack of awareness. I think there’s a lack of understanding, a lack of communication, and conversations about what mental illness is and how it shows up. … the stigma that comes from it and experiences often don’t have understanding, [unless] you have witnessed it with a family member, and try to learn and understand… I think some more education, more understanding, and some more awareness around those illnesses, and how they affect people, and how they affect families. …important to have the conversations and to normalize it, if she had cancer or a heart attack, we would talk about it, and so we need to talk about it [mental illnesses]’
- Participant 11: ‘Very comfortable [discussing my brother’s diagnosis]. I [participant] go to my close friends for support. Again, because …it’s been such a chronic problem sometimes, I feel like I don’t want to burden my friends with the same problems over and over, because nothing changes’
- Participant 18: ‘And a lot of my [participant] close friends don’t know just because it’s not my place to necessarily inform them, but there are some of my in- laws side of the family that do know, so I’ve shared that information with them. Just because they are within the family and have been in situations where they’ve been around him [participant’s brother] more frequently than my own friends. So, I wouldn’t say I’m comfortable sharing his information with my friends, or his friends …’
- Participant 6: ‘Still, I [participant] am very uncomfortable with extended family members. But I think with friends, we may talk about it with those really close friends. … I don’t think that I would want to talk about it with anybody, say at work, like a colleague, or something like that. And even like with some family members, you know when they see something in the news about a mother losing a child or something and they’ll make a comment, and I might need to remind them that my mother lost a child, and they don’t seem to equate it in the same way’
- Participant 3: ‘…I [participant] was kind of embarrassed 30 years ago because I didn’t know anything about it [mental illness], and I really thought that he would recover at some point, even just recovering or get better. But now …I don’t go out of my way to tell people in conversation, but I think I am pretty open about it. I think it’s much better now than it was 30 years ago. I think I can say to people I have one sibling, and this is his situation. And he’s not able to work, and I’m his caregiver and so it’s not like I hide it’
- Participant 26: ‘…I [participant] have gotten a lot less shy about telling people. I used to say anything at the beginning. I just didn’t want to admit that my brother had paranoid schizophrenia, … because I thought about stigma. I thought this was a heavy thing for people to know. I didn’t want them to feel uncomfortable talking to me because they know that I have this situation in my family. but I’ve become very mature of fact about it over the decades. And now I just say, you know, I have a brother who lives with paranoid schizophrenia. He lives in a special housing situation for people who were on the street, and …, if they just take their medication, then I pipe up and I say that’s not the way. …I will make an effort to educate people, and say it’s not just about taking medication. Taking medication is not a complete solution for anybody who or everybody who has mental health challenges…’
- Participant 19: ‘No, I [participant] think generally her perfect family seems to have an open mind and our understanding. I think we’ve all become more informed throughout the process, so perhaps in the early stages, we had some misconceptions about it [mental illness]. But I think generally there isn’t a stigma. I know that my son, who is an adult, has concerns about the medication [or] the drugs. And I don’t know, there may be some stigma associated with that’
- Participant 15: ‘I [participant] think as a family we’ve been educated about this [diagnosis] and we know my parents know as well. I don’t think so. Like I said, after we got educated …we understand now the kind of help she needs from us…’
- Participant 26: ‘I [participant] would say nobody in our family stigmatizes my brother because mom was still alive when he was first diagnosed, and she spent quite a bit of time with him... My sister, a nurse practitioner, was very good about dealing with him. My other sister who suffered from mental illness herself was very open and took him to live with her for 10 years. I would… but he and I were closest growing up. We’ve continued to communicate, and I haven’t had as much direct contact with him. Our in-laws and children have been fine and comfortable with him. He came home for our mom’s funeral and people approached him and talked to him …’
- Participant 24: ‘I [participant] think changing what we see in the media and the representation that we see…. I think having buried normal boring people with mental illness, being more represented, that it’s just an idea that if you have a mental illness, you just kind of live a normal life, and you aren’t crazy or evil,... I think especially with the more serious diagnoses like schizophrenia, we have a fear of it that it’s somehow scary or bad when in fact it’s just people living their lives. In fact, in my first year, I took a psychology course and I realized how wrong I was about what I thought schizophrenia was until I was taught, because that was just my perception from media, growing up. That is, just the idea that people with mental illnesses are normal and kind of boring and aren’t inherently evil or want to hurt other people. …’
- Participant 3: ‘…I [participant] think certainly if I’m in a situation and I hear people saying things that are not true about people with mental illness, I will definitely speak up and tell them that what they’re saying is not valid. …It’s very surprising to me when in this day and age when people say things that show that they have stigma against people with mental illness … if you understood mental illness, you would realize that people would never choose that life intentionally. … I don’t know what the solution is, I guess that just talking about it right in the media and in the healthcare settings [and] health classes that the kids get in high school to teach them about what mental illness looks like and that they shouldn’t discriminate against it…’
- Participant 13: ‘…I [participant] would call them out on it. And I would try and educate them on what I know about depression and about what I know about her mental illness …’
- Participant 1: ‘…I [participant] had contacted her [participant’s younger sister] and said that we have to deal with mum’s apartment and belongings… If you would like to come over and assist me,...so she [participant’s younger sister] did come and she brought her husband and one of her sons, … and upon entering the apartment, they all had something to say. And I warned them… she [mom] had been in psychosis for the last 2 years, so her home is in disarray. And police enter the apartment, and all things that come out of your mouth need to have a lens of empathy. She was sick and didn’t have the support. So, I needed to adjust this really firmly with my sister that I don’t want to hear snide remarks that she was not part of the last 20 years of her life. And you can’t just walk in here and start dishing out remarks …’
- Participant 19: ‘… I [participant] think they [policymakers] could do a better job. I think it tends to maybe fall more to the not-for-profits and to do more of that public education around it [mental illnesses]. …I think that my daughter has received very good care and it’s never been a case where she hasn’t been able to access it, and you know she can actually see crisis team, she can access and I think that’s been working well for her but I think there’s lots of people though that maybe have challenges with access so I think that’s where policymakers could improve the situation.’
- Participant 11: ‘…there’s an older generation of individuals like my [participant] brother who have been living with a diagnosis for so long. And they’re caught between worlds and people who are more newly diagnosed, or are getting the benefit of the changes to some of our approaches to care, which are becoming more compassionate and becoming more trauma[tic] and violence-informed. But people like my brother have gone a little bit too far into being discriminated against and mistreated, and excluded to the point where there are no real programs that can help him…’
- Participant 25: ‘…, informing people about the full spectrum of what to expect during medication treatment [and] how to come off… is key. And I think people ought to have more choice about how they live their lives, if they choose to live with diversity, you know, diverse ways of thinking, such as having hallucinations and delusions to some degree, is not a crime, and people have a right to live that way…’
- Participant 25: ‘… challenging to figure out how to deliver services. Better and more person-centered, but I think that there’s room for more creativity in how those policies roll down the programs they run. And I think we definitely need more funding put towards people’s mental well-being. I think people need access to key publicly paid-for short and brief interventions’
- Participant 24: ‘They [policy-makers] can do more. There should be more free quality support that’s accessible’
- Participant 30: ‘…I do believe that policy-makers are accepting it [mental illnesses], but I feel like it’s a struggle for people who have a mental illness to get by because of disability. Checks are very limited, so if my brother didn’t have a house that my mother bought him, he would be in a little [or] would be the worst thing’
- Participant 18: ‘…I think it is crucial, as well as having a proper foundation of health care. I think a lot of the times when you go into emergency departments, for example, the mental health capacities they’re just overflowing with people who require assistance, … Do you see these people who are experiencing homelessness and who also experienced a mental health illness, you often associate it with that mental health illness automatically means that you’re going to be homeless which isn’t necessarily the case. So, I really think proper support is crucial in our healthcare system, and I think the wait times for some of those programs, and for initial assessments, are just really heartbreaking…’

A member of the *PLOS One* Editorial Board reviewed the updated quotes and stated that the participants’ quotes have been rephrased with minimal additions, and that the conclusions of [1] remain supported.

## References

[1] Adu J, Oudshoorn A, Anderson K, Marshall CA, Stuart H. Negotiating familial mental illness stigma: the role of family members of persons living with mental illnesses. PLoS One. 2024;19(9):e0311170. doi: 10.1371/journal.pone.0311170 39348379 PMC11441641

